# Oocyte-somatic cells interactions, lessons from evolution

**DOI:** 10.1186/1471-2164-13-560

**Published:** 2012-10-19

**Authors:** Cathy Charlier, Jérôme Montfort, Olivier Chabrol, Daphné Brisard, Thaovi Nguyen, Aurélie Le Cam, Laurent Richard-Parpaillon, François Moreews, Pierre Pontarotti, Svetlana Uzbekova, Franck Chesnel, Julien Bobe

**Affiliations:** 1 INRA, UR1037 LPGP Fish Physiology and Genomics, Campus de Beaulieu, Rennes, F-35000, France; 2 INRA, UMR85 Physiologie de la Reproduction et des Comportements, CNRS 7247, Université de Tours, Nouzilly, F-37380, France; 3 INRA, SIGENAE/PEGASE, Rennes, F-35000, France; 4 CNRS, Université de Rennes 1, UMR6290, Rennes, F-35000, France; 5 LATP UMR-CNRS, Aix-Marseille University 7353, Evolution Biologique et Modélisation, Marseille, F-13331, France

## Abstract

**Background:**

Despite the known importance of somatic cells for oocyte developmental competence acquisition, the overall mechanisms underlying the acquisition of full developmental competence are far from being understood, especially in non-mammalian species. The present work aimed at identifying key molecular signals from somatic origin that would be shared by vertebrates.

**Results:**

Using a parallel transcriptomic analysis in 4 vertebrate species - a teleost fish, an amphibian, and two mammals - at similar key steps of developmental competence acquisition, we identified a large number of species-specific differentially expressed genes and a surprisingly high number of orthologous genes exhibiting similar expression profiles in the 3 tetrapods and in the 4 vertebrates. Among the evolutionary conserved players participating in developmental competence acquisition are genes involved in key processes such as cellular energy metabolism, cell-to-cell communications, and meiosis control. In addition, we report many novel molecular actors from somatic origin that have never been studied in the vertebrate ovary. Interestingly, a significant number of these new players actively participate in Drosophila oogenesis.

**Conclusions:**

Our study provides a comprehensive overview of evolutionary-conserved mechanisms from somatic origin participating in oocyte developmental competence acquisition in 4 vertebrates. Together our results indicate that despite major differences in ovarian follicular structure, some of the key players from somatic origin involved in oocyte developmental competence acquisition would be shared, not only by vertebrates, but also by metazoans. The conservation of these mechanisms during vertebrate evolution further emphasizes the important contribution of the somatic compartment to oocyte quality and paves the way for future investigations aiming at better understanding what makes a good egg.

## Background

The importance of the somatic compartment in oocyte developmental competence (i.e. the oocyte ability to be fertilized and subsequently develop into a normal embryo) acquisition has been stressed in many vertebrate species. In mammals, bidirectional communications between oocyte and surrounding somatic cells are required for meiotic maturation and acquisition of oocyte competence [1-4]. Follicular cells control oocyte development and maturation *via* specific molecular signals including different LH-receptor activated signaling pathways [5-7]. In prophase I-arrested oocytes, meiosis inhibitory signals from somatic cells pass through gap junctions between the oocyte and surrounding somatic cells [8]. Meiotic maturation is associated with inactivation of these gap junctions, and this process is evolutionary conserved between mammals and *C. elegans*[9]. In teleost fish, when meiosis resumption can be triggered directly by the maturing steroid hormone acting at the oocyte level, the resulting metaphase II oocytes sometimes exhibit poor developmental competence. In contrast, when meiosis resumption is induced by gonadotropin hormones acting upstream (i.e. at the somatic level of the ovarian follicle), a much higher developmental competence is often observed [10]. Several bodies of evidence further indicate in several fish species that, in addition to the well documented roles in meiotic maturation, somatic compartments of the ovarian follicle play an important role to allow full oocyte developmental competence acquisition [11-13]. A number of studies have subsequently demonstrated that many genes were actively expressed in somatic follicular layers during final oocyte maturation including genes coding for steroidogenic enzymes, paracrine factors, gonadotropin receptors, and cell-to-cell communication-related genes such as connexins (see [14-16] for review). Less is known about the contribution of follicular somatic cells to oocyte developmental competence in amphibians as the focus has mostly been made on intra-oocyte mechanisms underlying growth and meiotic maturation. The role of granulosa cells in the steroidogenic control of oogenesis is nevertheless well established and, similarly to fish, communications between oocyte and adjacent somatic cells are established through gap junctional complexes in a gonadotropin-dependent manner. This gap junction coupling has been shown to facilitate vitellogenin uptake by the oocyte [17]. Likewise, maternal *Xenopus* activin which is involved in mesoderm induction during early embryogenesis is initially produced by ovarian follicular cells and transferred along with vitellogenin to the growing oocytes [18,19]. Interestingly, the oocyte was shown to influence steroidogenesis in surrounding granulosa cells, thus indicating a dialog between follicular and somatic compartments of the ovarian follicle [20].

Despite the known importance of somatic cells for oocyte developmental competence acquisition, the overall mechanisms underlying full developmental competence acquisition are far from being understood. This is especially true in non-mammalian species. In addition, most existing studies have been devoted to a single species. To date, no comprehensive view of the contribution of molecular actors from somatic origin to oocyte developmental competence acquisition is available. The present works thus aimed at identifying key molecular signals, from somatic origin, that would be shared by evolutionary distant vertebrate species in order to offer an evolutionary perspective on oocyte developmental competence acquisition in vertebrates.

Using a parallel transcriptomic analysis of the somatic cells surrounding the oocyte in 4 vertebrate species - a teleost fish, an amphibian, and two mammals - at key steps of developmental competence acquisition, we identified a surprisingly high number of orthologous genes exhibiting similar expression profiles among the 3 tetrapods and among the 4 vertebrates including a significant subset of novel molecular actors from somatic origin, some of them being also shared by *Drosophila*.

## Results

### Identification of differentially expressed genes in the 4 studied species

A microarray analysis was performed using somatic follicular cells surrounding the oocyte in 4 vertebrate species (mouse, cow, *Xenopus*, and trout) at 3 similar key stages of oocyte developmental competence acquisition: developmentally incompetent or poorly competent prophase I oocytes (NC1 oocytes), developmentally competent prophase I oocytes (C1 oocytes), and developmentally competent metaphase II oocytes (C2 oocytes). Corresponding data were deposited in Gene Expression Omnibus (GEO) database in a SuperSeries under the reference GSE36617.

In rainbow trout, 1341 probes were found to be differentially expressed between at least 2 of the studied stages ( Additional file 1). Theses probes were blasted on existing rainbow trout public contigs. A BlastX analysis (e-value < 10^-3^) was subsequently performed using identified rainbow trout contig sequences in order to identify corresponding zebrafish (*Danio rerio*) sequences. The corresponding unique 706 full-length zebrafish protein sequences were subsequently used for further OrthoMCL analysis to identify groups of orthologous genes among the differentially expressed genes of each species ( Additional file 2).

In *Xenopus laevis*, 4220 probes were found to be differentially expressed between at least 2 of the studied stages ( Additional file 1). Among those 4220 probes, 2802 unique *Xenopus laevis* RefSeq sequences could be identified and kept for further OrthoMCL analysis ( Additional file 2).

In cow, 3867 probes were found to be differentially expressed between at least 2 of the studied stages ( Additional file 1). Among those 3867 probes, 2833 unique *Bos taurus* RefSeq sequences could be identified and kept for further OrthoMCL analysis ( Additional file 2).

In the mouse, 7087 probes were found to be differentially expressed between at least 2 of the studied stages ( Additional file 1). Among those 7087 probes, 5971 unique *Mus musculus* RefSeq sequences could be identified and kept for further OrthoMCL analysis ( Additional file 2).

### Orthologous genes exhibiting similar expression profiles in at least 2 species

#### ***Orthologous genes exhibiting similar expression profiles in the 2 studied non-mammalian species***

**Figure 1:**
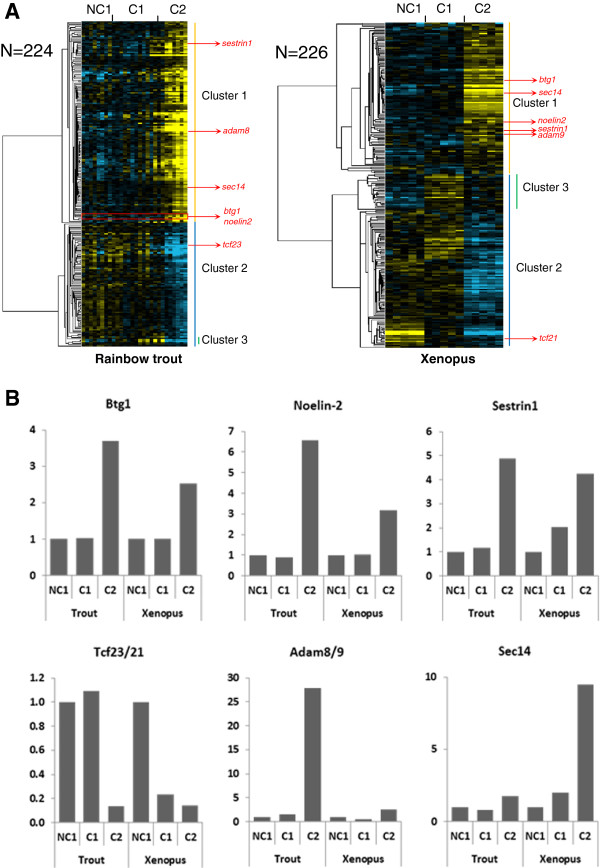
**Differentially expressed orthologous genes exhibiting a conserved expression profile in rainbow trout and *Xenopus.*** (**A**) Supervised clustering of expression profiles in the somatic layers of the ovarian follicle in rainbow trout and *Xenopus* during oocyte developmental competence acquisition: developmentally incompetent or poorly competent prophase I oocytes (NC1), developmentally competent prophase I oocytes (C1), and developmentally competent metaphase II oocytes (C2). For each species, the number of differentially expressed orthologous genes is indicated. (**B**) The expression profiles of specific genes are shown.

A total of 195 OrthoMCL groups (i.e. groups of orthologous genes [21]) were found in which at least 1 rainbow trout gene and 1 *Xenopus* gene exhibited a similar – up or down - differential expression between at least 2 among the 3 studied oocyte developmental stages ( Additional file 3). In rainbow trout, 224 genes belonged to an OrthoMCL group in which at least 1 *Xenopus* gene had a similar expression profile (Figure 1A). In *Xenopus*, 226 genes belonged to an OrthoMCL group in which at least 1 rainbow trout gene had a similar expression profile (Figure 1A). In both species, two main expression clusters could be identified that corresponded to up- and down-regulated genes at C2 stage, respectively. In rainbow trout, in which all growing follicles develop simultaneously and progressively during reproductive season, the expression profiles observed were more progressive than in *Xenopus* and were correlated to the progressive follicular differentiation stage within each studied group. In contrast, expression profiles in *Xenopus* exhibited a strict stage-dependent profile. In addition to the 2 main expression clusters characterized by a marked up- (cluster 1) or down-regulation (cluster 2) in C2 follicles, a smaller cluster characterized by an up-regulation in C1 follicles and a subsequent down-regulation in C2 follicles was found in both species (cluster 3, Figure 1A). As an example of the conserved expression profiles between trout and *Xenopus*, anti-proliferative B-cell translocation gene 1 (Btg1) exhibited a sharp 3.7-fold and 2.5-fold over expression at C2 stage in trout and *Xenopus*, respectively (Figure 1B). Similarly, Noelin-2 exhibited a 6.6- and 3.2-fold overexpression at C2 stage in trout and *Xenopus*, respectively. The expression profile of Sestrin1 was also characterized by a strong up-regulation at C2 stage, even though the up-regulation appeared more progressive throughout oocyte competence acquisition, especially in *Xenopus*. For some other genes, the differential expression observed at C2 stage was more pronounced in one of the two species as illustrated for Adam8/9 and Sec14. Among the genes exhibiting a stepwise down-regulation in the somatic cells surrounding the oocyte throughout the process of oocyte competence acquisition, a strong down-regulation was observed for the genes belonging to the Tcf21/Tcf23 group of orthologous genes (Figure 1B). 

Among all the genes exhibiting a differential expression in both non-mammalian species, a significant enrichment was observed in genes involved in proteasome and extra-cellular matrix (ECM)–receptor interaction pathways ( Additional file 4).

### Orthologous genes exhibiting similar expression profiles in the 2 studied mammalian species

**Figure 2:**
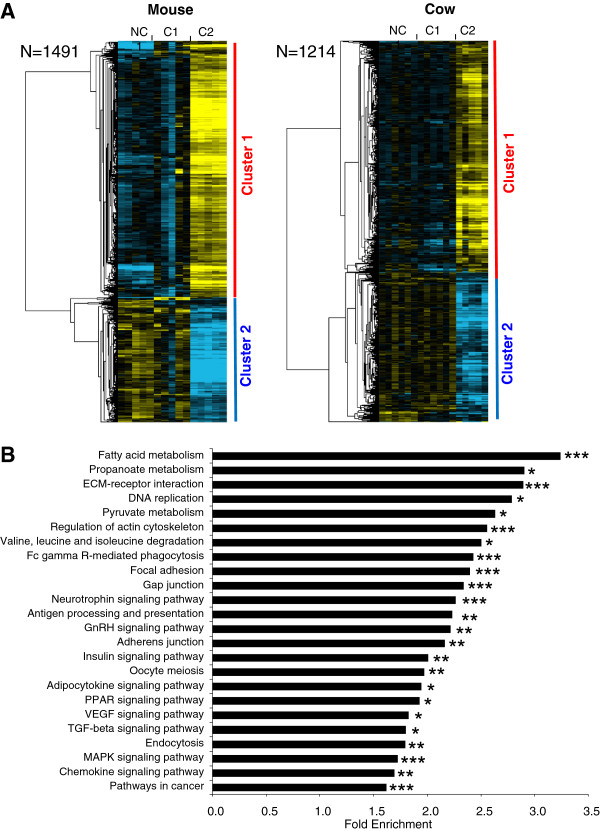
**Differentially expressed orthologous genes exhibiting a conserved expression profile in mouse and cow.** (**A**) Supervised clustering of expression profiles in the somatic layers of the ovarian follicle in mouse and cow species during oocyte developmental competence acquisition: developmentally incompetent or poorly competent prophase I oocytes (NC1), developmentally competent prophase I oocytes (C1), and developmentally competent metaphase II oocytes (C2). For each species, the number of differentially expressed orthologous genes is indicated. (**B**) Gene ontology and KEGG pathways enrichment score in clusters 1 and 2. Stars denote the p-values: * p<0.05; ** p<0.01; *** p<0.001.

A total of 1103 OrthoMCL groups were found in which at least 1 gene of the 2 species exhibited a differential expression between at least 2 among the 3 studied oocyte developmental stages ( Additional file 5). In the mouse, 1491 genes belonged to an OrthoMCL group in which at least 1 bovine gene had a similar profile (Figure 2A). In cow, 1214 genes belonged to an OrthoMCL group in which at least 1 mouse gene had a similar profile (Figure 2A). In the 2 species, the supervised clustering analysis resulted in the identification of 2 main gene clusters corresponding to a differential – up or down - expression in somatic cells surrounding C2 oocytes. Among all common differential genes in mammals, the most significant enrichment was observed in genes involved in energy metabolism such as fatty acid, pyruvate, and propanoate metabolism as well as insulin or adipocytokine signaling (Figure 2B). Were also enriched a number of genes involved in the regulation of focal adhesion, gap-junctions, actin cytoskeleton, and oocyte meiosis (Figure 2B). Moreover, numerous genes taking a part in PPAR, VEGF, MAP kinases and TGFbeta signaling were also enriched in both species ( Additional file 4).

### Orthologous genes exhibiting similar expression profiles in the 3 studied tetrapod species

**Figure 3:**
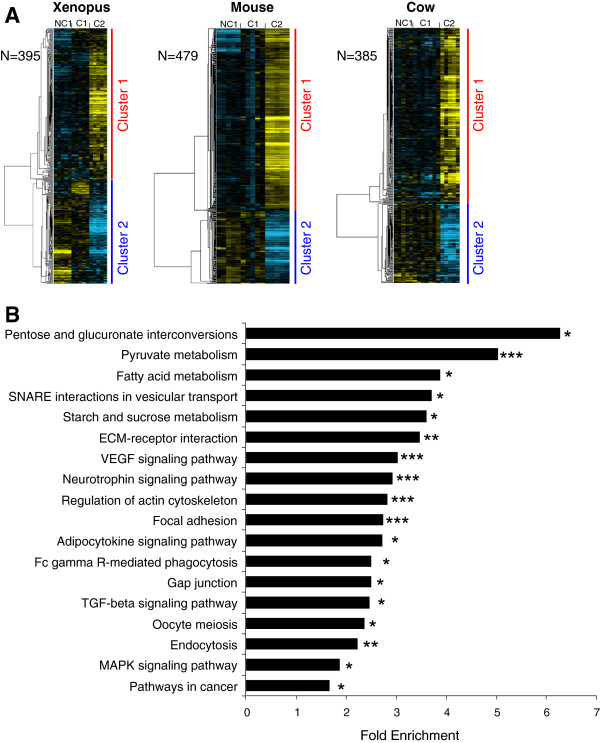
**Differentially expressed orthologous genes exhibiting a conserved expression profile in *Xenopus*, mouse, and cow.** (**A**) Supervised clustering of expression profiles in the somatic layers of the ovarian follicle in *Xenopus*, mouse, and cow species during oocyte developmental competence acquisition: developmentally incompetent or poorly competent prophase I oocytes (NC1), developmentally competent prophase I oocytes (C1), and developmentally competent metaphase II oocytes (C2). For each species, the number of differentially expressed orthologous genes is indicated. (**B**) Gene ontology and KEGG pathways enrichment score. Stars denote the p-values: * p<0.05; ** p<0.01; *** p<0.001.

A total of 340 OrthoMCL groups were found in which at least 1 gene of the 3 species exhibited a differential expression between at least 2 among the 3 studied oocyte developmental stages ( Additional file 6). In *Xenopus*, 395 genes belonged to an OrthoMCL group in which at least 1 gene of the 2 other tetrapod species had a similar expression profile (Figure 3A). In the mouse, 479 genes belonged to an OrthoMCL group in which at least 1 gene of the 2 other tetrapod species had a similar profile (Figure 3A). In cow, 385 genes belonged to an OrthoMCL group in which at least 1 gene of the 2 other tetrapod species had a similar profile (Figure 3A). In the 3 species, the supervised clustering analysis resulted in the identification of 2 main gene clusters corresponding to a differential – up or down - expression in somatic cells surrounding C2 oocytes. In both clusters, a significant enrichment in genes associated with specific biological processes, molecular functions, and pathways could be identified. Among the common genes differentially expressed in the 3 tetrapod species, the most significant enrichment was observed for genes involved in energy metabolism (carbohydrates and fatty acids conversions). We also observed a significant enrichment in genes involved in VEGF signaling, TGF-beta signaling, adipocytokine signaling, MAPK signaling, oocyte meiosis, focal adhesion, gap junctions and cytoskeleton regulation (Figure 3B and Additional file 4).

**Figure 4:**
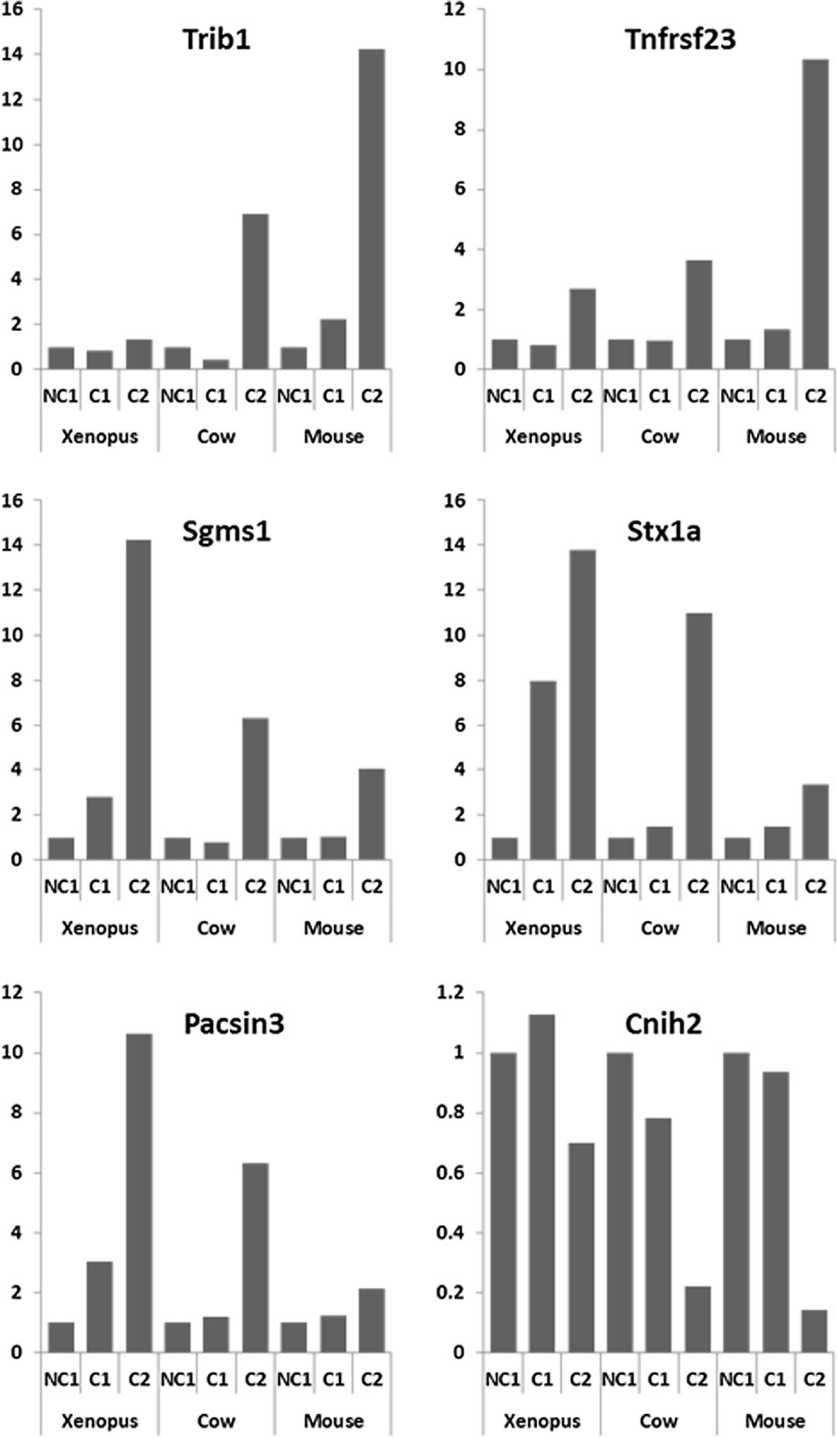
**Expression profiles of specific orthologous genes in *Xenopus*, mouse, and cow.** The microarray expression profiles of specific gene in the somatic cells surrounding the oocyte during competence acquisition are shown. The mean expression is shown for the following: developmentally incompetent or poorly competent prophase I oocytes (NC1), developmentally competent prophase I oocytes (C1), and developmentally competent metaphase II oocytes (C2).

Among the genes that were markedly differentially expressed in the 3 tetrapod species, an OrthoMCL group corresponding to the Tribbles gene family was identified. Hence, a dramatic over expression of Trib1 at C2 stage was observed in mouse and cow while the overexpression was more limited, even though significant, in *Xenopus* (Figure 4). Similarly a conserved overexpression was observed at C2 stage in the 3 species for the OrthoMCL groups corresponding to tumor necrosis factor receptor superfamily member 23 (Tnfrsf23), sphingomyelin synthase 1 (Sgms1), syntaxin 1A (Stx1a), and protein kinase C and casein kinase substrate in neurons 3 (Pacsin3) (Figure 4). In addition, genes exhibiting a marked down-regulation at the C2 stage could also be observed. In mice and cow, a dramatic down-regulation of cornichon homolog 2 (Cnih2) was observed while a milder decrease was observed in *Xenopus* (Figure 4).

### Orthologous genes exhibiting similar expression profiles in the 4 studied species

**Figure 5:**
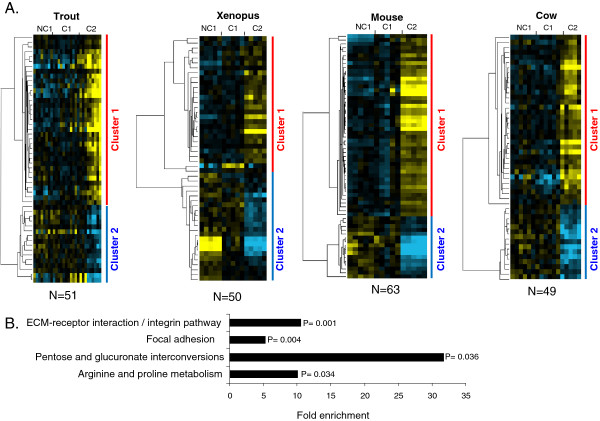
**Differentially expressed orthologous genes exhibiting a conserved expression profile in rainbow trout, *Xenopus*, mouse, and cow. A**. Supervised clustering of expression profiles in the somatic layers of the ovarian follicle in rainbow trout, *Xenopus*, mouse, and cow species during oocyte developmental competence acquisition: developmentally incompetent or poorly competent prophase I oocytes (NC1), developmentally competent prophase I oocytes (C1), and developmentally competent metaphase II oocytes (C2). For each species, the number of differentially expressed orthologous genes is indicated. **B**. Gene ontology enrichment score in clusters 1 and 2 (p<0.05).

A total of 42 OrthoMCL groups were found in which at least 1 gene of the 4 species exhibited a conserved differential expression between at least 2 of the 3 studied oocyte developmental stages ( Additional file 7). In rainbow trout, 51 genes belonged to an OrthoMCL group in which at least 1 gene had a similar profile in the 3 other species (Figure 5A). In *Xenopus*, 50 genes belonged to an OrthoMCL group in which at least 1 gene had a similar profile in the 3 other species. In the mouse, 63 genes belonged to an OrthoMCL group in which at least 1 gene had a similar profile in the 3 other species. In cow, 49 genes belonged to an OrthoMCL group in which at least 1 gene had a similar profile in the 3 other species. In the 4 species, the supervised clustering analysis resulted in the identification of 2 main gene clusters corresponding to a differential – up or down - expression in somatic cells surrounding C2 oocytes (Figure 5A). Four significantly enriched pathways were identified among the differentially expressed othologous genes common to the 4 species (Figure 5B and Additional file 4). As shown, they include genes involved in the regulation of focal adhesion (such as genes encoding actin filaments), ECM-receptor interaction (such as genes encoding for different collagens and integrins) and metabolism (such as genes of aldo-keto reductase family, nitric acid synthases, and spermidine/spermine N1-acetyltransferase 1).

**Figure 6:**
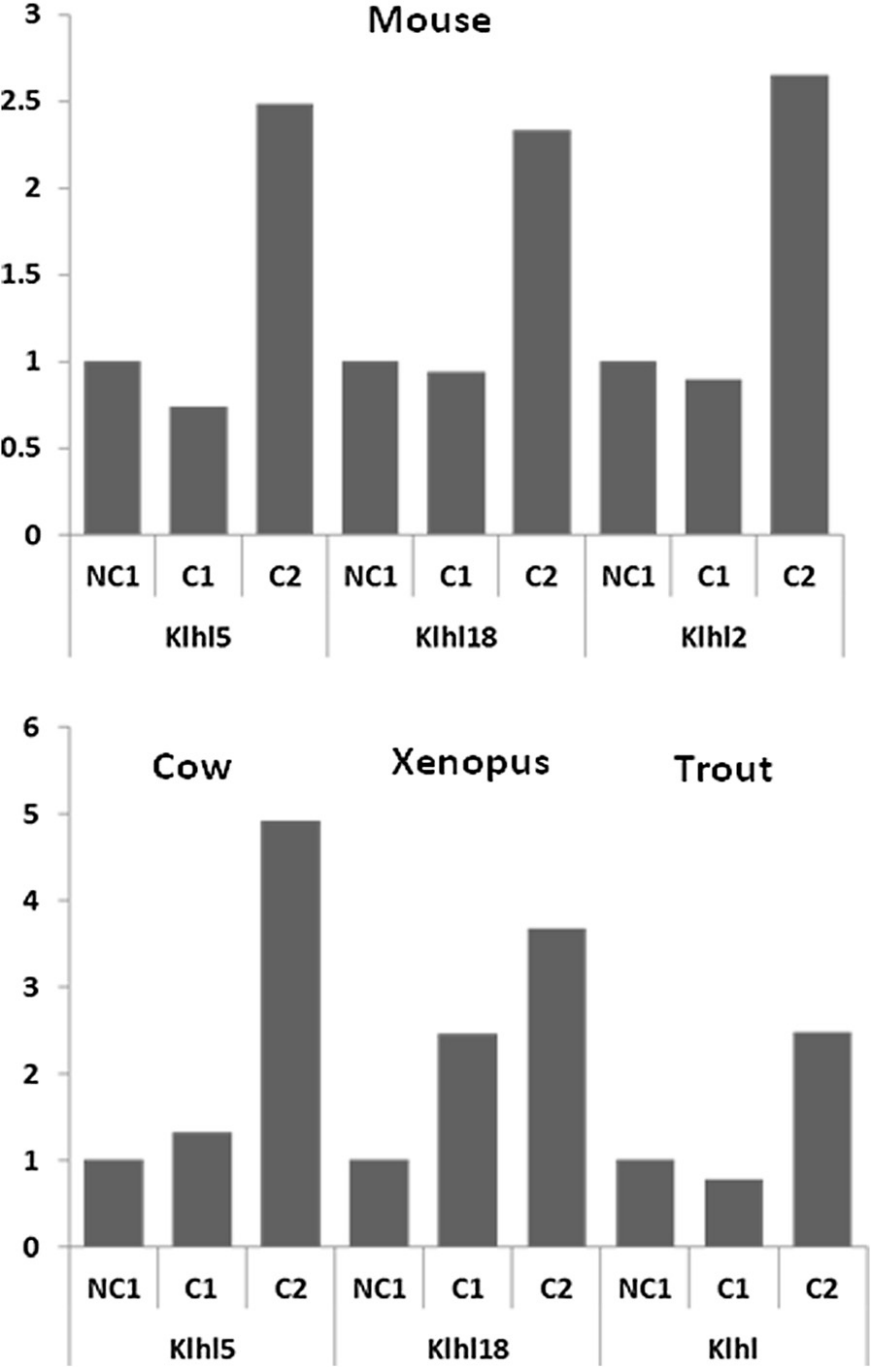
**Conserved expression profiles of genes of the Klhl gene family in somatic follicular cells during oocyte developmental competence acquisition.** Expression profiles in the somatic layers of the ovarian follicle in the 4 species during oocyte developmental competence acquisition: developmentally incompetent or poorly competent prophase I oocytes (NC1), developmentally competent prophase I oocytes (C1), and developmentally competent metaphase II oocytes (C2).

**Figure 7:**
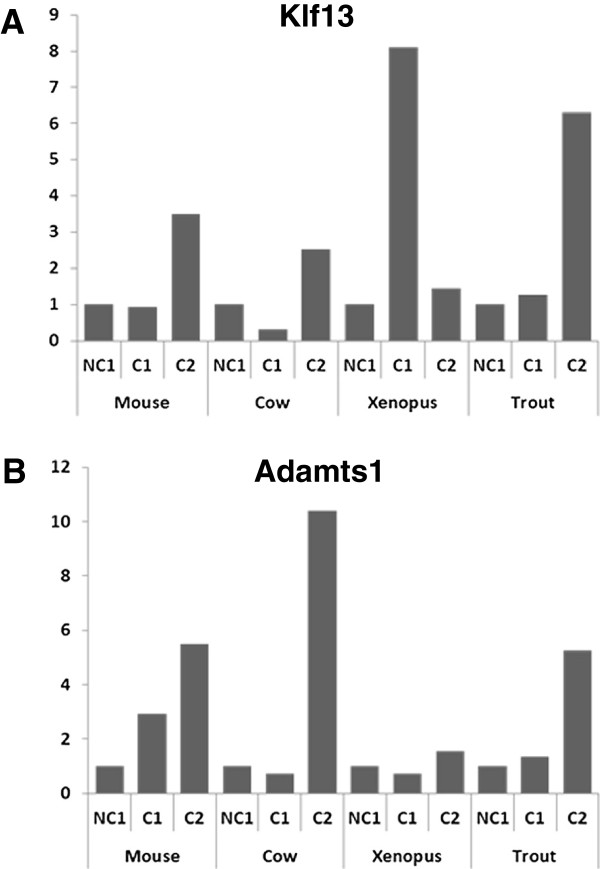
**Conserved expression profiles of Adamts1 and Klf13 genes in somatic follicular cells during oocyte developmental competence acquisition.** Expression profiles in the somatic layers of the ovarian follicle in the 4 species during oocyte developmental competence acquisition: developmentally incompetent or poorly competent prophase I oocytes (NC1), developmentally competent prophase I oocytes (C1), and developmentally competent metaphase II oocytes (C2).

Among the 42 OrthoMCL groups in which similar expression profiles were found in the 4 species, the following groups showed the most remarkable profiles. An OrthoMCL group corresponding to the Kelch family was identified in which several genes were differentially expressed (Figure 6). In the mouse, the genes Klhl5, Klhl18, and Klhl2 exhibited very similar expression patterns with a sharp increase at C2. In cow, a very similar expression pattern was observed for Klhl5 while Klhl18 exhibited a more progressive over-expression in *Xenopus*. In rainbow trout, even though the exact identity of the Klhl-related gene remained unclear, the pattern was highly similar to that of mouse and cow. An OrthoMCL group corresponding to Kruppel-like factor 13 (Klf13) was identified in which a similar differential expression profile was observed for the 4 species (Figure 7A). In all species the expression at C2 stage was higher than at NC1 stage. A burst in Klf expression was however observed in *Xenopus* at C1 stage, suggesting that overexpression occurred earlier in this species. Similarly, a clear increase in the expression of Adamts1 gene was observed in the 4 species (Figure 7B).

### QPCR validation

In order to methodologically validate our microarray-based analysis, the expression profiles of a subset of genes of interest, including up- and down-regulated genes presented in Figures 1, 4, and 7, were monitored using QPCR ( Additional files 8 and 9). The profiles obtained using QPCR confirmed the differential expression observed using microarrays.

## Discussion

In the present study we were able to identify orthologous genes exhibiting similar expression profiles in the somatic cells surrounding the oocyte during developmental competence acquisition in vertebrates. Our results rely on genome-wide transcriptome analyses carried out, in parallel, in four different vertebrate species using (i) a similar technology and (ii) similar key steps of developmental competence acquisition. The transcriptome analysis was followed by an OrthoMCL analysis to specifically identify orthologous genes exhibiting similar expression profiles. To our knowledge such an approach has never been used to investigate the oogenetic process in evolutionary distant vertebrate species.

### A surprisingly high number of orthologous genes exhibiting evolutionary-conserved expression profiles

We identified a surprisingly high number of orthologous genes exhibiting similar expression profiles at key steps of oocyte-developmental competence in different vertebrate species. This is especially true in mice and cow in which ~1500 and ~1200 orthologous genes were identified, respectively. This is also the case in tetrapod species in which ~400 orthologous genes were found to be differentially expressed in the 3 species among a total of 4000–8000 differentially expressed genes in each species. It has to be stressed that the highest number of differentially expressed genes (~8000) was found in the mouse in which the number of spotted probes was twice as high as in the 2 other species. Together, this suggests that approximately 10% of the differentially expressed genes have orthologs in the 2 other tetrapod species that behave similarly during oocyte developmental competence acquisition. It should be noted that the percentage of orthologous genes with similar profiles is remarkably high when considering the major differences in follicle ultrastructure and physiological regulations that exist between amphibians and mammals. This suggests a strong conservation -and possibly a crucial role - of the corresponding molecular mechanisms during vertebrate evolution despite the presence of an antrum in the mammalian ovarian follicle and the absence of antrum in non-mammalian vertebrates. In agreement with this hypothesis is the significant enrichment in genes involved in cellular energy metabolism, cell-to-cell communications, and meiosis observed in the three tetrapod species and the two mammalian species. Among the four vertebrate species studied herein, a much more limited set of common differentially expressed genes could be identified, in comparison to the large number of common genes shared by the three tetrapod species or by the two mammalian species. This can largely be explained by the evolutionary distance between the species, especially between rainbow trout and mammals. Several methodological reasons also exist that have possibly reduced the number of common genes identified. These reasons are mostly due to the lack of genome sequence availability in rainbow trout and its consequences on the design of the oligonucleotides and on subsequent *in silico* analysis. Despite these difficulties, we were able to highlight genes and pathways involved in oocyte developmental competence acquisition in the four vertebrate species (Figures 5, 6 and 7). In consistency with data obtained in tetrapods and mammals, our results stress the importance of the nutritive role of the somatic cells surrounding the oocyte and of the mechanisms involved in extracellular matrix formation. In addition, more than 200 common genes were identified in rainbow trout and *Xenopus* as highlighted in the result section (Figure 1).

In the present study, we have deliberately chosen to highlight some of the genes exhibiting similar profiles in the somatic compartment during oocyte developmental competence acquisition in several vertebrate species. Many groups of orthologous genes (i.e. OrthoMCL groups) were however identified that could not be discussed here due to space limitations. All corresponding expression profiles and OrthoMCL information are however available (see additional files) and can be searched for specific genes or mechanisms. We believe that this unique dataset will be very useful to investigators in the fields of reproductive biology and evolutionary biology. Similarly, the large species-specific datasets generated in the present study can be further analyzed by other investigators to address species-specific questions.

### Mammals

As indicated above, our approach has led to the identification of more than 1200 orthologous genes that deserve further analysis. In the present study, we have deliberately chosen to focus our analysis on common genes shared by tetrapods or vertebrates in agreement with the main objectives of our study. We will however briefly highlight in this section some of the identified genes differentially expressed in both mouse and cow in order to stress the biological relevance of our approach and to clearly demonstrate the methodological validity of our results.

### Extra-cellular matrix remodeling linked to ovulation

Cathepsin S (Ctss) and Cathepsin B (Ctsb) are lysosomal cysteine proteinases involved in degradation of extracellular matrix. Their transcripts (OG_10260 and OG_10174, respectively) were over-expressed in cumulus complexes (CC) of mature (C2) oocytes in mice and cow in comparison to CCs surrounding developmentally incompetent oocytes (NC1). In fact, Ctss and Ctsb genes were reported as markers of oocyte developmental competence in cow and their expression in CCs of metaphase-II oocytes was shown to be high in comparison to immature oocytes [22]. Both cathepsins were also detected and localized in mice ovary [23]. Secreted acidic cysteine rich glycoprotein (Sparc) is a matricellular protein expressed during tissue morphogenesis and reorganization, and we observed an increase of transcript level (OG_10175) between NC1 and C2 stages in both mice and cow. In agreement with our observations, high expression levels of Sparc were previously reported in mice cumulus oophorus [24]. In bovine CCs, the upregulation of Sparc was related to cumulus expansion in response to growth factors complementation during IVM [25].

### Ligands and receptors of the developing follicle

Follistatin (Fst) is involved in inhibition of FSH release, and was also found to be expressed in mice and bovine OCs [26,27]. Our transcriptomic analysis revealed the high expression of Fst gene (OG_11886) in immature CCs in both species and a significant decrease after maturation. Similarly, the transcript levels of Anti-Mullerian hormone (Amh, OG_11935) and one of its receptors AMH type II receptor (Amhr2; OG_11318) dramatically decreased in CCs upon cow and mouse oocyte maturation (C2 stage compared to NC1 and C1 stages). Such an expression profile of the hormone has already been recently described in cow ovary [28] as well as in many other mammalian species (sheep, rat, primates, and humans - reviewed by [29]). To our knowledge, Amhr2 expression pattern has not been studied in cow ovaries but in rat as well as in mouse, this receptor was shown to be mainly expressed in granulosa cells of preantral and early antral follicles [30,31]. Similar observations can be made for the FSH receptor (OG_10792) for which a down-regulation was observed herein at C2 stage in both species as previously observed at least in cow ovaries [32].

### Intra-cellular cell signaling

Phosphodiesterases (PDEs) regulate intracellular cyclic nucleotide concentration by converting cyclic nucleotides into 5' nucleotides. Their activity was found in mice and bovine granulosa/cumulus cells [33,34] and inhibition of PDE3/PDE4 activity blocks meiosis in bovine oocytes [35]. In our study, several genes encoding PDEs were found to be similarly expressed in mice and bovine. Thus, mRNA level of Pdea4 and Pde6d (OG_10100 and OG_11911) increased during maturation, and that of Pde5a and Pde7a (PL_10380530) decreased. Similarly, ERβ transcript (OG_11926) is down regulated between C1 and C2 stage in bovine and mouse cumulus in the present study in agreement with previous reports demonstrating a predominant expression of this ER isoform in small rodent preovulatory follicles [36,37] and a down-regulation by gonadotropins [36]. Such a regulated pattern of expression was also reported in bovine follicles [32].

### Tetrapods

Among the genes exhibiting a conserved profile during oocyte developmental competence acquisition, some were previously known to play an important role as illustrated hereafter. This further validates the approach used in the present study. For instance, growth hormone receptor (Ghr) has been shown to be expressed in ovarian follicles in many vertebrate species and involved in folliculogenesis, particularly by promoting secondary follicle growth and antrum formation in mammals as shown in cases of Ghr deficiency in cow [38] and in mice [39] (see [40] for review). In the present study, the corresponding transcript (OG_10283) was highly expressed in NC1 and or C1 stage granulosa cells and was down-regulated in C2 stage in the 3 tetrapod species. These observations are in full agreement with previous reports in cow, mice and even fish. Indeed, a study conducted at the protein level in rainbow trout showed that ovarian growth hormone receptor concentration was high during the early phases of follicular development and subsequently decreased during oocyte and follicular growth to reach a minimal value during oocyte maturation [41].

In addition to genes known to participate in ovarian functions, our study also shed light on a large number of molecular actors for which a role in ovarian physiology was previously unsuspected or poorly investigated. This is for instance the case of a decoy TNF receptor gene up-regulated at C2 stage (Tnfrsf23; OG_10201, Figure 4). The importance of the TNF system in folliculogenesis is well known and many TNF ligands and “classical” receptors are differentially regulated during oogenesis in a wide variety of vertebrate species [42-44]. Although the precise role of Tnfrsf23 in the mammalian ovary remains uncharacterized, it is thought to be a decoy TNF receptor [45]. Decoy TNF receptors have the ability to bind TNF ligands without inducing cell death. Interestingly, alternatively spliced variants of Tnfrsf23 are expressed in the chicken ovary [46] and a decoy TNF receptor was found to be differentially expressed in the fish ovary during final oocyte maturation [47]. An increased expression of a set of genes, including sphingomyelin synthase 1 (Sgms1), syntaxin 1A (Stx1a), and Pacsin3, which are implicated to a different extent in exo/endocytosis and protein trafficking, was also observed in our study, particularly at C2 stage (Figure 4). To the best of our knowledge, none of these genes has ever been reported to play a role in ovarian follicle/oocyte development. Sgms1, by regulating cellular levels of ceramide and diacylglycerol, can affect the proliferation/apoptosis balance and it has only been shown to participate in TNF-mediated apoptosis in Chinese Hamster Ovary cells [48]. Stx1a is rather expressed in neurons while the expression of other syntaxins, such as Stx4a or Stx7/avalanche, has been reported in human granulosa cells or in drosophila follicle cells, respectively [49,50]. Finally, Pacsin3 besides its role in vesicular transport, membrane dynamics, and cytoskeleton organization also participates in addressing some ion channels and glucose transporters to the plasma membrane [51]. In the present study, down-regulation of a cornichon-related gene (Cnih2 also known as Cni1) expression was evidenced in the somatic compartment during oocyte competence acquisition in the 3 tetrapod species (Figure 4). Cornichon (Cni) proteins have been studied in *Drosophila* and Yeast, Cni being involved in *Drosophila* embryo polarization [52]. Four genes exist in vertebrates (named *Cnih*, *Cnih2*, *Cnih3*, and *Cnih4* in the mouse). Recent evidence suggested that metazoans cornichon proteins have a similar subcellular localization and regulate transport of TGFα family ligands [53]. The transcripts of Cnih are highly abundant in full grown oocyte and unfertilized egg in mice. A role in ovarian follicular regulation has, however, never been reported in any vertebrate species to date. Interestingly, Cni plays an important role in *Drosophila* oogenesis and more specifically in a signal transduction pathway that transmits information between the germline cells and the somatic follicle cells of the ovary [54]. Together, these observations suggest an important, yet unsuspected, role of the Cnih family in the somatic compartment of the follicle during oocyte competence acquisition in tetrapods.

Finally, up-regulation of Tribbles homolog 1 (Trib1) was evidenced herein during oocyte maturation (i.e. between C1 and C2 stages) in surrounding somatic cells (Figure 4). Trib1 belongs to a family of genes (Tribbles 1, 2, 3, and 4) that has been implicated in the development of many diseases including cancers (reviewed by Kiss-toth [55]); for instance, Trib1 is involved in the onset of acute myeloid leukemia (AML) by regulating C/EBP transcription factor levels and activity through a MAPK (MEK1/ERK)-dependent pathway [56], but is also associated with hyperlipidemia [57]. Interestingly, Yamamoto et al. [58] have observed that Trib1-deficient drosophila and mouse females are infertile; in drosophila, such a phenotype is likely due to the fact that Trib1 has been shown to regulate migration of a cluster of ovarian follicle (epithelial) cells, called border cells, leading to micropyle formation required for oocyte fertilization. In this case, Trib1 function would also involve a post-translational regulation of the drosophila C/EBP homologue, slbo [59]. In mammals, the mechanisms underlying Trib1 participation in ovarian folliculogenesis and oocyte development remain however to be elucidated.

Together, our results demonstrate that tetrapods share a large number of common molecular actors from somatic origin that have similar expression profiles during developmental competence acquisition. This suggests that many conserved mechanisms exist in the ovary that are required or important for the acquisition of a full developmental competence. We also showed that many of the new players presently identified in the context of oocyte competence acquisition in our study were previously unstudied in the ovary and deserve further investigations. Finally, our results reveal that some of the important molecular actors from somatic origin found in tetrapods are also shared by some non-vertebrate metazoan species. This suggests that some of the key signals from somatic origin involved in oocyte developmental competence acquisition would be shared, not only by vertebrates, but also by metazoans.

### Vertebrates

Among the four vertebrate species studied, a total of 42 groups of orthologous genes could be identified that shared similar expression profiles. As for tetrapods, our study shed light on novel molecular players from somatic origin. This is for instance the case of the kelch family for which different members are differentially expressed, depending on the species. The Kelch protein was initially discovered in *Drosophila* in which it is known to play an important role during oogenesis and more specifically in the communications between somatic cells and oocyte. Such communications are required for the development of a normal oocyte and mutation of *kelch* leads to female infertility [60]. The kelch protein family is a large multigenic family and over 30 Kelch-like proteins (KLHL) exist in humans. A comprehensive picture of the entire family is however currently lacking in vertebrates and little is known about the role of Kelch proteins during oogenesis. Our results, evidencing a conserved regulation of Kelch gene expression in the somatic compartment are thus extremely original and suggest an evolutionary-conserved important role of kelch proteins in the development of a normal oocyte in insects and vertebrates. Our results are also consistent with the abundant expression of *KLHL5* previously reported in human ovary [61].

Similarly, the Krüppel-like factor (KLF) is also a large multigenic family named after the *Drosophila* Krüppel protein and 17 *KLF* genes can be found in the human genome [62,63]. The KLFs are transcription factors that regulate a wide variety of biological processes including proliferation, growth, development, survival, and responses to external stress (see [63] for review). Their role in reproductive functions has, in contrast, received very little attention. In the present study, we report the up-regulation of Klf13 mRNA expression during oogenesis in the four species. This pattern is consistent with the previous report of Klf13 mRNA expression in swine granulosa cells [64]. In addition, several Klf genes were reported to be differentially expressed in the ovarian follicle under normal or pathological conditions [65-67]. Together, this suggests an important role of the KLF family in vertebrate oogenesis and a conserved pattern of Klf13 expression in the somatic compartment during intrafollicular competence acquisition.

Finally, we have chosen to highlight the up-regulation of genes belonging to the Adamts1 (a disintegrin and metalloproteinase with thrombospondin like repeats 1) OrthoMCL group at C2 stage in the four vertebrate species studies. In mammals, Adamts1 plays an important role during ovulation in mammals to prepare the oocyte for fertilization [68]. Such role in the inflammatory-like ovulatory process is in agreement with the profiles reported here in mice and cow. In non-mammalian vertebrates, the mechanisms underlying ovulation are less documented. The inflammatory nature of the ovulatory process has, however, been established in fish [69]. The present work strongly suggests that the important role played by Adamts1 in mammals to prepare the oocyte for fertilization would also be found by non-mammalian vertebrates.

Together, the observations made in the 4 vertebrate species studied further support the results obtained in the 3 tetrapod species that suggest that some of the key signals from somatic origin involved in oocyte developmental competence acquisition would be shared, not only by vertebrates, but also by metazoans.

## Conclusions

In the present work, we have developed a vertebrate-wide transcriptomic analysis of the contribution of the somatic compartment at key stages of oocyte developmental competence acquisition. Our approach has led to the identification of a large number of species-specific differentially expressed genes and a surprisingly high number of orthologous genes exhibiting similar expression profiles in evolutionary distant species among tetrapods and among vertebrates. Our study demonstrates the usefulness and power of a parallel transcriptomic analysis performed in evolutionary distant vertebrate species to identify evolutionary-conserved mechanisms.

Among the evolutionary conserved participants in developmental competence acquisition are genes involved in key processes such as cellular energy metabolism, cell-to-cell communication, and meiosis control. In addition, our results shed light on novel molecular signals from somatic origin during vertebrate oogenesis, some of them being also shared by non-vertebrate species. Together, our results suggest an important role of some conserved molecular mechanisms from somatic origin in oocyte developmental competence during the oogenetic process in vertebrates. Our results also strongly suggest that some of the key players from somatic origin involved in oocyte developmental competence acquisition would be shared, not only by vertebrates, but also by metazoans. The conservation of these mechanisms during vertebrate evolution further emphasizes the important contribution of the somatic compartment to oocyte developmental competence acquisition during oogenesis and paves the way for future investigations aiming at better understanding what makes a good egg.

## Methods

### Animal care and tissue collection

Investigations and animal care were conducted in compliance with French and European regulation on the care and use of laboratory animals. Procedures were approved by the Agricultural and Scientific Research Government Committee (approval A37801) in accordance with the guidelines for Care and Use of Agricultural Animals in Agricultural Research and Teaching and by the Rennes Ethics Committee for animal use (approval R-2010-FC-01). As indicated above, the aim of the present work was to sequentially study the molecular signals of somatic origin at critical steps of oocyte development in 4 vertebrate species. To achieve this objective, the somatic cells surrounding the oocyte were sampled in the 4 studied species at the following stages: developmentally incompetent or poorly competent prophase I oocytes (NC1 oocytes), developmentally competent prophase I oocytes (C1 oocytes), and developmentally competent metaphase II oocytes (C2 oocytes).

### Rainbow trout (*Oncorhynchus mykiss*)

Twenty eight adult rainbow trout females (*Oncorhynchus mykiss*) from an autumn-spawning strain were obtained from the INRA/PEIMA fish farm (Sizun, France) and transferred into a re-circulated water system. Before sampling, fish were deeply anaesthetized in 0.03% (v/v) 2-phenoxyethanol in water and subsequently euthanized Ovaries were then dissected out of the body cavity under sterile conditions. In rainbow trout, all vitellogenic follicles (i.e. 2000–3000 per kg of body weight) develop simultaneously in the ovary. It is thus necessary to sample ovarian follicles from different females to obtain follicles at successive steps of development. To do so, ovaries were sampled at successive stages of ovarian development throughout reproductive season. For each sampled ovary, the maturational competence of follicle-enclosed oocytes was assessed using an *in vitro* oocyte maturation assay as previously described [70]. NC1 follicles were sampled from late vitellogenic females in which meiosis resumption could not be triggered using LH rich partially purified gonadotropin (PPG) [71]. C1 follicles were sampled from post-vitellogenic females in which oocyte maturation can be triggered using PPG. As previously demonstrated, the oocytes obtained following gonadotropin-induced oocyte maturation can be subsequently fertilized [11]. C2 follicles were obtained from maturing females in which follicle-enclosed oocyte had resumed meiosis. In addition, 2 post-vitellogenic females in which follicle-enclosed oocytes spontaneously resumed meiosis in culture without any hormonal stimulation were incorporated into the C2 group. For all studied stages, individual ovarian follicles were dissected as previously described in rainbow trout [11,70], and the follicular envelopes removed using forceps. NC1, C1, and C2 follicles were sampled from 6, 14, and 8 different females, respectively. Samples originating from several females of each studied stages were fixed and used for subsequent histological analysis to demonstrate the presence of both granulosa and theca layers in the samples used for microarray analysis.

### *Xenopus* (*Xenopus laevis*)

Ovarian pieces were surgically removed from six *Xenopus* anaesthetized adult females (*Xenopus laevis*), purchased from NASCO (Fort Atkinson, WI, USA). In *Xenopus*, the different stages of follicular development (I-VI; [72]) are simultaneously present in the ovary, in contrast to rainbow trout. For the microarray analysis, NC1 follicles were sampled at stage IV, while C1 follicles were sampled at stage VI as described below. Eighty to 100 *Xenopus* stage IV (stage IV, 800-μm diameter) and around 50 stage VI (stage VI, 1200-μm diameter, in prophase I of meiosis) ovarian immature follicles were manually isolated in OR2 buffer (83 mM NaCl, 2.5 mM KCl, 1 mM CaCl_2_, 1 mM MgCl_2_, 1 mM Na_2_HPO_4_, 5 mM HEPES, pH 7.4) from ovarian pieces of each female.

In order to obtain follicle-enclosed oocytes in metaphase II of meiosis (C2 follicles) from the same females, gonadotropin-induced maturation was induced *in vitro* by further incubating immature stage VI follicles for 15 h at room temperature in OR2 buffer supplemented with 40 IU/mL of human chorionic gonadotropin (hCG; Organon, Puteaux, France). After the incubation period, the meiotic competence of the oocytes was assessed by direct observation of germinal vesicle breakdown (GVBD) under a stereomicroscope reflected by the appearance of a white spot on the animal pole of the follicle-enclosed oocytes. At all sampled stages, the envelopes of follicular cells (i.e. granulosa and theca) surrounding the oocytes were then manually recovered after a 30-min treatment with collagenase (type 1A, 275 IU/ml; Sigma-aldrich) in calcium-free OR2 buffer, rinsed in PBS. Cell pellets were snap-frozen in liquid nitrogen and stored at −70°C until RNA extraction.

### Cow (*Bos taurus*)

NC1 cumulus-oocytes complexes (COCs) were recovered by aspiration of 3–6 mm ovarian follicles from 6 individual prepubertal 3–6 months calves. COCs recovery was performed post mortem in a commercial slaughterhouse. C1 COCs were obtained from 6 adult cows using the same procedure. C2 COCs, enclosing developmentally competent oocytes, were recovered by ovum pick up (OPU) from large pre-ovulatory follicles of 5 cycling, non-lactating Holstein cows from INRA local farm setting following a previously described FSH-based super-ovulation protocol [2]. Briefly, after post-mortem ovarian aspiration, only COCs with several layers of compact cumulus cells surrounding the oocyte were selected to collect immature cumulus cells (CC) samples. To obtain *in vivo* matured COCs, adult cows were synchronized by progestagen ear implants (Crestar® method; Intervet, France) and then superovulated with porcine FSH/LH Stimufol preparation (SPRL Reprobiol, Liege, Belgium), given as two daily injections over four days in a decreasing schedule (total dose of 400 μg/animal in 7 doses). On the 5^th^ dose of FSH, a prostaglandin F2α injection was given and OPU was performed 60 h after prostaglandin injection. COCs were recovered by OPU from follicles with a diameter greater than 8 mm by transvaginal aspiration under the control using ultrasound echography. Only COCs with expanded cumulus enclosing mature oocyte were selected. Cumulus cells were mechanically separated from the oocytes rapidly after collection, then precipitated by centrifugation and then kept frozen at −80°C until RNA extraction. The meiotic status of mature oocytes was determined by chromatin labelling with Hoechst33342 (Sigma, 1 μg/mL), followed by microscopic observation.

### Mouse (*Mus musculus*)

NC1 and C1 unexpanded cumulus-oocytes complexes (COCs) were isolated from 5 groups of B6CBAF1/J mice (4–5 per group; Charles River Laboratories, Larbresle cedex France) at 3- and 8-week of age, respectively. COCs were removed from ovaries by puncturing large follicles in M2 culture medium (Sigma-Aldrich, St-Quentin Fallavier, France). C2 mature expanded COCs were obtained from 8-week-old mice (5 groups of 4 females) first primed for 48 h with a single peritoneal injection of 5 IU pmsg (Merck chemicals, Nottingham, UK) followed by 16-hr ovulatory stimulus of 5IU hCG (Schering Plough, Courbevoie, France). In the latter case, COCs flushed from the excised oviduct ampullas were transferred into M2-droplets containing 300 μg/ml of hyaluronidase (Sigma-Aldrich) for a few seconds to disperse granulosa cells. In all cases, media containing granulosa cells after oocyte denudation and elimination were centrifuged at 900 *g* and cell pellets rinsed in PBS before snap-freezing in liquid nitrogen and storage at −70° until RNA extraction.

### RNA extraction and microarray analysis

Total RNA was extracted as previously described [70] using Tri-reagent and following manufacturer’s instruction. The total RNA yield was estimated using a Nanodrop ND-1000 spectrophotometer (Labtech, Palaiseau, France) and RNA integrity was checked with an Agilent Bioanalyzer (Agilent Technologies, Massy, France). Rainbow trout gene expression profiling was conducted using an Agilent 4X44K high-density oligonucleotide microarray designed by Salem and coworkers. (GEO platform # GPL6018) [73]. Labeling and hybridization steps were performed following the “One-Color Microarray-Based Gene Expression Analysis (Quick Amp labeling)” Agilent protocol. Briefly, for each sample, 300–350 ng of total RNA was amplified and labeled using Cy3-CTP. Yield (>1.65 μg cRNA) and specific activity (> 9 pmol of Cy3 per μg of cRNA) of Cy3-cRNA produced was checked with the Nanodrop. 1.65 μg of Cy3-cRNA was fragmented and hybridized on a sub-array. Hybridization was carried out for 17 hours at 65°C in a rotating hybridization oven prior to washing and scanning with an Agilent Scanner (Agilent DNA Microarray Scanner, Agilent Technologies, Massy, France) using the standard parameters for a gene expression 4×44K oligoarray (5 μm and 20 bits). Data were then obtained with the Agilent Feature Extraction software (10.5.1.1) according to the appropriate GE protocol (GE1_105_Dec08). Before analysis, saturated spots, non-uniform spots and spots not significantly different from background (k=5) were flagged using the Agilent GeneSpring GX software (10.0.2). Probes were considered valid when corresponding spots remained present in at least 80% of the replicates of each experimental condition after the flagging procedure. Data were subsequently scale-normalized using the median value of each array.

For *Xenopus*, Bovine, and Mouse the microarray analysis was carried out as described above for rainbow trout and using Agilent available designs (GEO platform # GPL11258, GPL9712, and GPL10333, respectively).

### Reverse transcription and real-time PCR analysis

In *Xenopus*, reverse transcription (RT) was performed from 500 ng of total RNA from somatic follicular cells originating from different individuals (N=4 for all groups) using 200 units of SuperScriptIII™ reverse transcriptase (Invitrogen, Cergy Pontoise, France) and 500 ng random hexamers (Promega) in a reverse transcription master mix containing 2 mM dNTPs, 50 mM Tris–HCl, 75 mM KCl, 3 mM MgCl2, 10 mM dithiothreitol, pH 8.3. In the mouse, RT was performed as described above using 350 ng of total RNA from cumulus cells (N=4 pools originating from different individuals for all groups). Twenty units of RNase inhibitor (RNasin, Promega) were added to the reaction. RNA and dNTPs were denatured for 5 min at 65°C, and then chilled on ice before addition of reverse transcription master mix. Reverse transcription was performed at 25°C for 7.5 min then at 50° for 1 h followed by a 15-min incubation step at 70°C. For both species, control reactions were run without reverse transcriptase and used as negative controls in the real-time polymerase chain reaction (PCR) study for all target genes. QPCR was done using the ABI PRISM 7000 real-time PCR system (Applied Biosystems, Foster City, USA). Reverse transcription products were diluted to 1/20 for both *Xenopus* and mouse. Triplicates were run for each RT product. Real-time PCR was performed using a real-time PCR kit provided with a SYBR Green fluorophore (Power SYBR Green Master Mix, Applied Biosystems). Primer concentration was 300 nM for 18S and 600 nM for all other genes (see Additional file 8 for primer sequences). The hot start enzyme was activated 10 min at 95°C, then the amplification was carried out using the following cycle: 95°C for 15 sec; 60°C for 1 min; 40 times. A pool of reverse transcribed RNA was serially diluted and used to calculate a standard curve. For all studied genes, 18S was used as internal standard to normalize the signal.

In rainbow trout, RT was performed using 1.5 μg of total RNA from follicular layers originating from different individuals (NC1, N=6; C1, N=17; C2, N=7). Quantitative real-time PCR (QPCR) analysis was carried out as previously described in this species with minor modifications [74]. In cow, RT was performed using 400 ng of total RNA from cumulus cells (N=5 pools originating from different individuals for all groups) and QPCR carried out as previously described with minor modifications [75].

### Data analysis

Statistical analysis was carried out in GeneSpring using a one factor ANOVA to identify differentially expressed genes among ovarian developmental stages. A Benjamini-Hochberg correction was applied (p-value<0.01). The identification of orthologous groups among 2, 3, or 4 of the studied species was performed using OrthoMCL [21,76] and only applied to the previously identified differentially expressed genes. Because no reference genome is available in rainbow trout, corresponding Refseq sequences were obtained from the zebrafish (*Danio rerio*) using a reciprocal best blast hit strategy. Corresponding zebrafish, *Xenopus*, mouse, and cow full length protein sequences were subsequently used for the identification of orthologous groups with OrthoMCL. The orthologous genes found in 2, 3, or 4 species were subsequently screened in order to identify genes of the same group of orthologs exhibiting a similar significant differential expression between at least 2 of the 3 assayed developmental stages (NC1, C1, and C2). In each species, clustering analysis was subsequently carried out using CLUSTER and TREEVIEW softwares as previously described [77]. Gene ontology (GO) analysis was carried out as follows. We performed GO enrichment analysis using the Database for Annotation, Visualization, and Integrated Discovery (DAVID) webservice ( http://david.abcc.ncifcrf.gov/) [78], which employs a Fisher's Exact Test. Briefly, the goal is to identify annotation terms and pathways significantly enriched in each gene set compared to a background list (here we used total genes list as background). Kyoto Encyclopedia of Genes and Genomes (KEGG) database was chosen to analysis of pathways enrichment scores. Corrected p-value cut-off of 0.05 was used to obtain functional categories and KEGG pathways for each analysis.

## Competing interests

The authors declare that they have no competing interests.

## Authors’ contributions

CC carried out the microarray study and the statistical analysis and participated in data analysis. JM participated in microarray analysis, *in silico* analysis and data mining. TN participated in rainbow trout sample collection and carried out corresponding RNA extraction and QPCR. DB carried out bovine sample collection, subsequent RNA extraction, and QPCR. LRP participated in *Xenopus* and Mouse sample collection and corresponding RNA preparation. AL participated in RNA preparation and microarray analysis. FM participated in the *in silico* analysis. OC and PP carried out the OrthoMCL analysis. JB, FC, PP, and SU conceived the study, participated in sample collection, microarray analysis, QPCR, data analysis and wrote the manuscript. All authors read and approved the final manuscript.

## Supplementary Material

Additional file 1 **Differentially expressed genes in trout, *Xenopus*, cow, and mouse.** For each species, probe name and corresponding oligo sequence are shown for all differentially expressed spots. The corresponding normalized expression values are given for all studied samples. For each sample, the corresponding group -developmentally incompetent or poorly competent prophase I oocytes (NC1), developmentally competent prophase I oocytes (C1), or developmentally competent metaphase II oocytes (C2)- is indicated. Data from different species are displayed in separate datasheets. All corresponding data were submitted to Gene Expression Omnibus database under # GSE36617. Click here for file

Additional file 2 **RefSeq accession numbers used for the OrthoMCL analysis.** For each species, the probe names and corresponding NCBI RefSeq accession numbers used for the OrthoMCL analysis are displayed. Data from different species are displayed in separate sheets of the file. For rainbow trout, the corresponding zebrafish protein accession numbers used in the analysis are shown. Click here for file

Additional file 3 **Orthologous trout and *Xenopus*genes exhibiting a similar expression profile.** For each OrthoMCL group of orthologous genes, corresponding RefSeq accession numbers and description are shown. Data from different species are displayed in separate datasheets. Click here for file

Additional file 4 **Pathways.** DAVID-defined KEGG pathways annotation categories for the lists of genes similarly expressed in non-mammalian species (A), mammals (B), tetrapods (C) and in all four analysed species (D). For each set of common differential genes, list of all genes present on mice AgilentV2 microarray was used as a background. Analysis reported the score and p-value of enrichment of GO terms related to different pathways in each genes set. For differentially expressed genes common to *Xenopus* and trout (A), a zebrafish and a *Xenopus laevis* background were used. Click here for file

Additional file 5 **Orthologous mouse and cow genes exhibiting a similar expression profile.** For each OrthoMCL group of orthologous genes, corresponding RefSeq accession numbers and description are shown. Data from different species are displayed in separate datasheets. Click here for file

Additional file 6 **Orthologous *Xenopus*, cow, and mouse genes exhibiting a similar expression profile.** For each OrthoMCL group of orthologous genes, corresponding RefSeq accession numbers and description are shown. Data from different species are displayed in separate datasheets. Click here for file

Additional file 7 **Orthologous trout, *Xenopus*, mouse and cow genes exhibiting a similar expression profile.** For each OrthoMCL group of orthologous genes, corresponding RefSeq accession numbers and description are shown. Data from different species are displayed in separate datasheets. Click here for file

Additional file 8 **QPCR primers.** The primer sequences are shown for all the genes analyzed using QPCR. For each OrthoMCL group, the accession numbers and primer sequences used in each species are shown. Click here for file

Additional file 9 **QPCR validation of specific gene expression profiles.** Expression profiles of specific genes in somatic follicular cells during oocyte developmental competence acquisition: developmentally incompetent or poorly competent prophase I oocytes (NC1), developmentally competent prophase I oocytes (C1), and developmentally competent metaphase II oocytes (C2). Expression values were normalized using 18S in rainbow trout, Xenopus and mice, and using RPL19 in cow. Expression profiles obtained in the microarray analysis are shown. (M). Click here for file
