# Supplementary material for: Oocyte-somatic cells interactions, lessons from evolution
Source: BMC Genomics. 2012 Oct 19;13:560. doi: 10.1186/1471-2164-13-560 (PMC3532176; doi:10.1186/1471-2164-13-560)
Supplement: Additional file 9 — QPCR validation of specific gene expression profiles. Expression profiles of specific genes in somatic follicular cells during oocyte developmental competence acquisition: developmentally incompetent or poorly competent prophase I oocytes (NC1), developmentally competent prophase I oocytes (C1), and developmentally competent metaphase II oocytes (C2). Expression values were normalized using 18S in rainbow trout, Xenopus and mice, and using RPL19 in cow. Expression profiles obtained in the microarray analysis are shown. (M). [file 1471-2164-13-560-S9.pptx]

## Slide 1
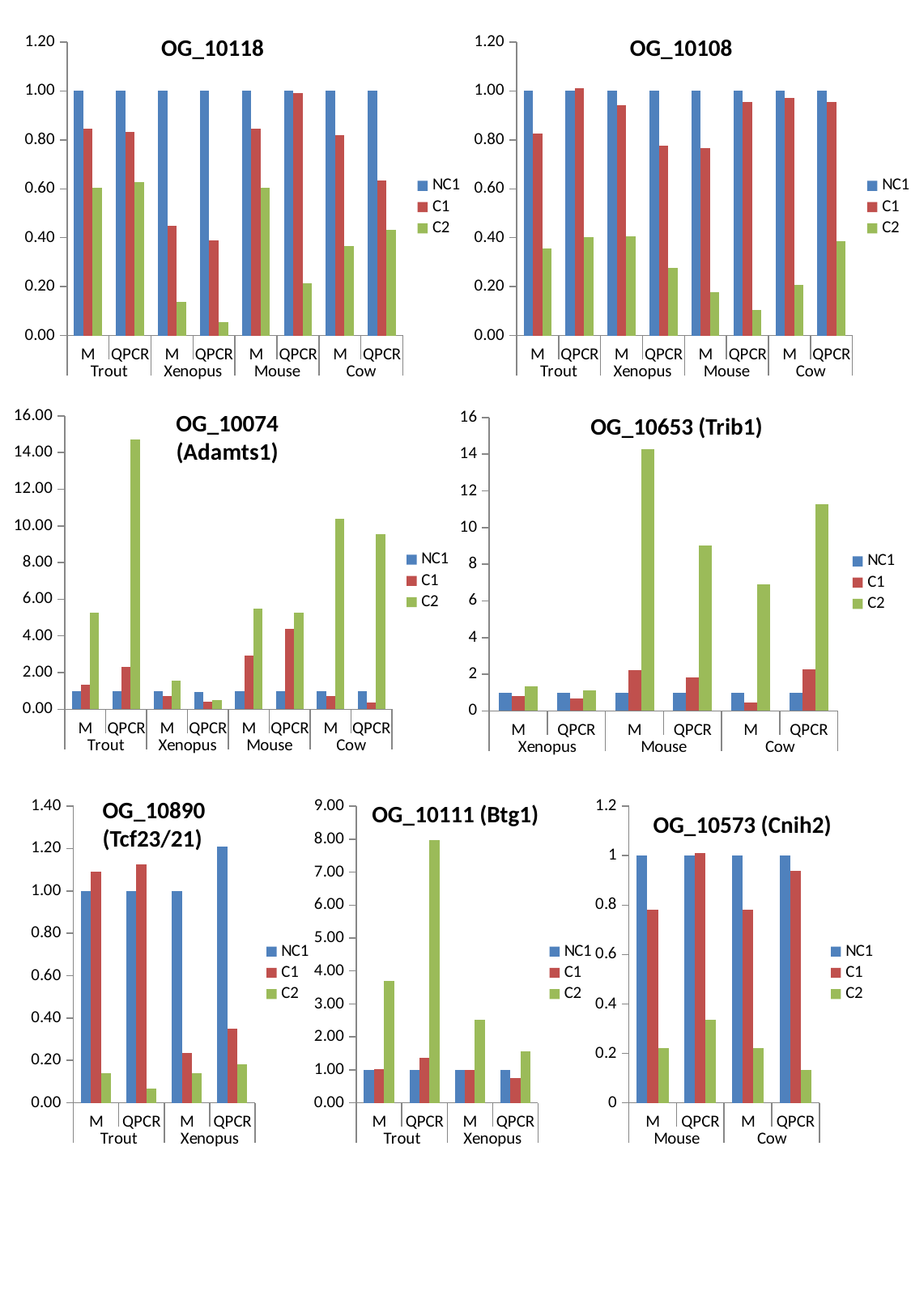

### Chart
| Category | NC1 | C1 | C2 |
|---|---|---|---|
| M | 1.0 | 0.8465547310584236 | 0.603662389228128 |
| QPCR | 1.0 | 0.8311547662177928 | 0.6274117181005714 |
| M | 1.0 | 0.4473734481626756 | 0.1373695598622268 |
| QPCR | 1.0 | 0.38888888888888884 | 0.05555555555555555 |
| M | 1.0 | 0.8465547310584236 | 0.603662389228128 |
| QPCR | 1.0 | 0.98989898989899 | 0.21212121212121213 |
| M | 1.0 | 0.8189300671279802 | 0.3660686369280992 |
| QPCR | 1.0 | 0.6354768431205158 | 0.4322367189019269 |OG_10118
### Chart
| Category | NC1 | C1 | C2 |
|---|---|---|---|
| M | 1.0 | 0.8275657542092494 | 0.3550280556441934 |
| QPCR | 1.0 | 1.01163744803731 | 0.4009332081192257 |
| M | 1.0 | 0.9433042038451894 | 0.4055627762477003 |
| QPCR | 1.0 | 0.7765957446808511 | 0.2765957446808511 |
| M | 1.0 | 0.7675506728216503 | 0.17588215281219877 |
| QPCR | 1.0 | 0.9552238805970149 | 0.10447761194029852 |
| M | 1.0 | 0.9719300565609574 | 0.20729388125578818 |
| QPCR | 1.0 | 0.9541529031089809 | 0.3863209260730575 |OG_10108
### Chart
| Category | NC1 | C1 | C2 |
|---|---|---|---|
| M | 1.0 | 1.3383377232485494 | 5.268720706043062 |
| QPCR | 1.0 | 2.288381123095932 | 14.732229188138902 |
| M | 1.0 | 0.7198862553444484 | 1.5437471122920052 |
| QPCR | 0.96 | 0.42 | 0.52 |
| M | 1.0 | 2.9370334237532587 | 5.495926975433954 |
| QPCR | 1.0 | 4.380281690140845 | 5.267605633802818 |
| M | 1.0 | 0.7323280180464626 | 10.391405412757129 |
| QPCR | 1.0 | 0.38381577034364733 | 9.563365113151159 |OG_10074 (Adamts1)
### Chart
| Category | NC1 | C1 | C2 |
|---|---|---|---|
| M | 1.0 | 0.8038442705130172 | 1.325113547782434 |
| QPCR | 1.0 | 0.6949152542372882 | 1.1186440677966103 |
| M | 1.0 | 2.2141617667766855 | 14.2641876156686 |
| QPCR | 1.0 | 1.8169014084507045 | 9.028169014084508 |
| M | 1.0 | 0.4447869914632465 | 6.886854624852234 |
| QPCR | 1.0 | 2.26 | 11.273894682232086 |OG_10653 (Trib1)
OG_10890 (Tcf23/21)
### Chart
| Category | NC1 | C1 | C2 |
|---|---|---|---|
| M | 1.0 | 1.092232041536892 | 0.13851052230391292 |
| QPCR | 1.0 | 1.1268897812781558 | 0.06605311220277105 |
| M | 1.0 | 0.23461239546552146 | 0.13922763346389502 |
| QPCR | 1.21 | 0.35 | 0.18 |
### Chart
| Category | NC1 | C1 | C2 |
|---|---|---|---|
| M | 1.0 | 1.0262270821885404 | 3.6959822491079013 |
| QPCR | 1.0 | 1.3743137251449553 | 7.98107963416603 |
| M | 1.0 | 1.0006279412703831 | 2.527436182863617 |
| QPCR | 1.0 | 0.7500000000000001 | 1.5555555555555558 |OG_10111 (Btg1)
### Chart
| Category | NC1 | C1 | C2 |
|---|---|---|---|
| M | 1.0 | 0.7819664212585737 | 0.2201846647435267 |
| QPCR | 1.0 | 1.0120481927710843 | 0.3373493975903615 |
| M | 1.0 | 0.7819664212585737 | 0.2201846647435267 |
| QPCR | 1.0 | 0.94 | 0.13226801497585028 |OG_10573 (Cnih2)
